# Analysis of Protein-Phenolic Compound Modifications Using Electrochemistry Coupled to Mass Spectrometry

**DOI:** 10.3390/molecules23020264

**Published:** 2018-01-29

**Authors:** Constanze Kallinich, Simone Schefer, Sascha Rohn

**Affiliations:** Institute of Food Chemistry, Hamburg School of Food Science, University of Hamburg, Martin-Luther-King-Platz 6, 20146 Hamburg, Germany; constanze.kallinich@chemie.uni-hamburg.de (C.K.); simone.schefer@studium.uni-hamburg.de (S.S.)

**Keywords:** adduct formation, protein interaction, electrochemical oxidation, phenolic compounds

## Abstract

In the last decade, electrochemical oxidation coupled with mass spectrometry has been successfully used for the analysis of metabolic studies. The application focused in this study was to investigate the redox potential of different phenolic compounds such as the very prominent chlorogenic acid. Further, EC/ESI-MS was used as preparation technique for analyzing adduct formation between electrochemically oxidized phenolic compounds and food proteins, e.g., alpha-lactalbumin or peptides derived from a tryptic digestion. In the first step of this approach, two reactant solutions are combined and mixed: one contains the solution of the digested protein, and the other contains the phenolic compound of interest, which was, prior to the mixing process, electrochemically transformed to several oxidation products using a boron-doped diamond working electrode. As a result, a Michael-type addition led to covalent binding of the activated phenolic compounds to reactive protein/peptide side chains. In a follow-up approach, the reaction mix was further separated chromatographically and finally detected using ESI-HRMS. Compound-specific, electrochemical oxidation of phenolic acids was performed successfully, and various oxidation and reaction products with proteins/peptides were observed. Further optimization of the reaction (conditions) is required, as well as structural elucidation concerning the final adducts, which can be phenolic compound oligomers, but even more interestingly, quite complex mixtures of proteins and oxidation products.

## 1. Introduction

Despite many years of research on protein-phenolic compound interactions, predictions of protein adduct formation between various food ingredients are still challenging and not yet satisfyingly solved [1]. Proteins provide reactive side chains that might act as nucleophiles for a Michael-type reaction. Here, especially amino, indole, and thiol groups seem to be pre-requisite reaction sites. Especially covalently bound phenolic compounds might significantly affect physicochemical protein properties such as the hydrophobicity/hydophilicity balance, correspondingly affecting the protein structure, as well the technofunctional properties (e.g., solubility). In addition, influences on the biological properties of proteins and enzymes, such as enzyme activity [2], nutritional protein quality [3], and changes in the allergenicity of the considered components, seem to be more or less obvious. For example, studying the allergenicity of milk proteins is still of certain interest, as it might be influenced during processing and dairy product development [4]. In this regard, one can think of products in which milk or whey fractions are combined with ingredients derived from fruits and vegetables that provide high concentrations of plant phenolic compounds (e.g., whey beverages on a fruit juice basis).

Due to their chemical structure, plant phenolic compounds are highly reactive and can be easily oxidized enzymatically and non-enzymatically [5]. Autoxidation can lead to dimers and even higher oligomers. The very complex brown polymers, so called melanins, are formed preferably during enzymatic oxidation (‘apples turn brown when they are cut’). At the beginning of such reactions, quinones are formed, which are considered to be key-elements of follow-up reactions [6]. Intermediates are usually part of polymerization, hydration, and disproportionation reactions [7]. The oxidation products can react with each other, interact with other compounds, and remain even reactive. Nevertheless, the complex reaction system of the phenolic compound leads to complex structures [5,8].

The reaction between phenolic compounds and proteins is even more complex. Reaction sites are differentially distributed all over the molecule (depending on the genetically-coded amino acid sequence). The state of oxidation of the phenolic compound might be different due to endogenous factors such as the pH value, temperature, etc. So far, protein derivatives have been synthesized (for characterization) very traditionally by incubating proteins and phenolic compounds under alkaline conditions [9]. However, this leads to a very crude mix of reaction products. Not surprisingly, the characterization of specific adducts and reaction sites is not yet satisfying. In many studies, only the extent of modification was studied. In a kind of indirect approach, free amino or thiol groups remaining after the reaction were determined photometrically [9]. In the meantime, several further analytical techniques, such as capillary electrophoresis, electrospray mass spectrometry, high-performance affinity chromatography, NMR spectroscopy, fluorescence quenching, and multi-spectroscopic methodologies have been developed to characterize protein-phenol interactions [1,10]. However, identification of specific binding sites of small molecules within a protein sequence (and the corresponding conformational position) is still challenging. Moreover, reaction kinetics have to be considered as well. In the past, it was often believed that a single oxidized phenolic compound reacts directly with a protein side chain, but it has to be also considered that the (oxidized) phenolic compounds might react initially with themselves, before reacting with the nucleophilic, sometimes sterically hindered protein side chains [11]. An important aim of this research topic is the development of methods for explaining the structural conditions of reaction products, assignments of binding sites, and reaction mechanisms. The development of new synthesis strategies in combination with a direct characterization using high resolution mass spectrometry offers new possibilities and technological advances in the analytical tasks mentioned.

The applicability of electrochemistry with regard to the investigation of the reactivity and coupling possibilities of different substance classes has already been successfully tested [12,13]. With regard to phenolic compounds, previous research has so far been able to demonstrate an electrochemical oxidation of caffeic acid by means of a flow-through system [14]. In contrast to the prevalent oxidability of some phenolic representatives like caffeic acid, there are also those phenolic compounds that are not suitable for a complete oxidation due to structural conditions and the redoxpotential necessary for a transformation. Those phenolic compounds comprise, for example, representatives that have structural elements such as methoxy or keto groups and only one hydroxyl group such as ferulic acid [15,16], in which the formation of semiquinones with different possibilities for follow-up reactions has to be considered, as well [5].

This present study focused on the comparison of the redox potential of various phenolic compounds and the characterization of oxidation products with regard to their ability to act as reaction partners for food proteins or peptides thereof using electrochemistry. It aimed to evaluate the different redox potentials for forming either monomeric, dimeric, or even oligomeric oxidation products and their reactivity towards peptides resulting from a tryptic digest of the whey protein alpha-lactalbumin.

## 2. Results

### 2.1. Electrochemical Oxidation of Phenolic Acids by Means of EC/ESI-MS

**Figure 1:**
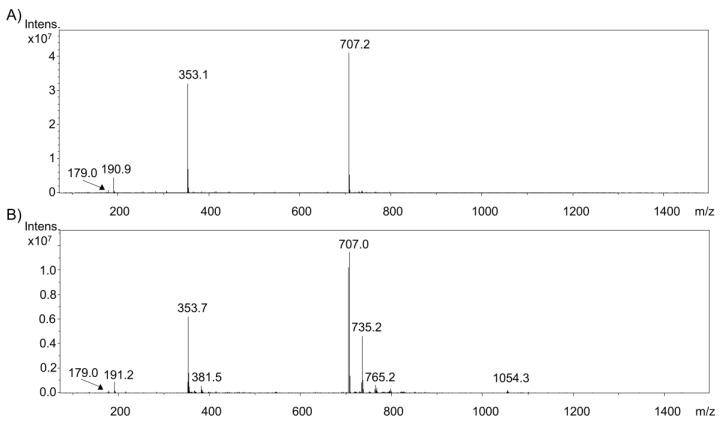
Full scan mass spectra of (**A**) intact chlorogenic acid and (**B**) oxidized chlorogenic acid using a potential of 1.8 V.

In the first step of this study, the applicability of an electrochemical oxidation for transforming phenolic compounds was evaluated. Exemplarily, the electrochemical oxidation of chlorogenic acid was optimized by varying the voltage from 0.3 V to 3 V in steps of 0.3 V using a boron-doped diamond electrode and a Roxy^®^ potentiostat (Antec Scientific, Zoeterwoude, The Netherlands). In the following mass spectrometry, the scan mode was used to identify the optimal potential concerning maximum recovery of the oxidation products. For chlorogenic acid, voltages from 0.6 V up to 1.8 V led to a bundle of oxidation products. Consequently, 1.8 V were chosen for the further experiments due to reproducible reaction product formation, diversity, and intensity. The resulting mass spectra are shown in Figure 1.

While the intensity of monomeric chlorogenic acid (*m*/*z* 353.7 [M − H]^−^) and dimeric derivatives (*m*/*z* 707.0 [M − H]^−^), as well as caffeic acid (*m*/*z* 179.0 [M − H]^−^) and quinic acid (*m*/*z* 191.1 [M − H]^−^), decreased during the oxidation, the ion intensity of three, so far unknown, compounds (*m*/*z* 381.5 [M − H]^−^; *m*/*z* 735.2 [M − H]^−^; *m*/*z* 765.2 [M − H]^−^), as well as the intensity of the trimeric derivative (*m*/*z* 1054.3 [M − H]^−^), increased significantly.

### 2.2. Comparison of the Oxidation of Various Phenolic Acids

To achieve comparability of the oxidability of the several phenolic compounds of interest, the electrochemical oxidation of four phenolic acids was evaluated. Besides chlorogenic acid, ferulic acid, caffeic acid, and sinapinic acid were oxidized electrochemically with a potential of 1.8 V analogously to chlorogenic acid. In all four reactions, a formation of dimeric and oligomeric products was observed. Considering caffeic acid and chlorogenic acid, the formation of oligomeric products was already detected at 0 V (caffeic acid: monomer (*m*/*z* 180.8 [M − H]^−^), dimer (*m*/*z* 363.0 [M − H]^−^), and trimer adduct (*m*/*z* 583.0 [M + 2Na − H]^−^). In addition, adduct and cleavage products are already recognizable: (caffeic acid: 3,4-dihydroxystyren (*m*/*z* 137.0 [M − H]^−^); chlorogenic acid: caffeic acid (*m*/*z* 179.0 [M − H]^−^), and quinic acid (*m*/*z* 191.1 [M − H]^−^). Upon application of 1.8 V, an increase of the oligomeric products (e.g., trimer of chlorogenic acid with *m*/*z* 1054.3 [M − H]^−^), and a decrease of the initial signals was observed. With regard to ferulic acid and sinapinic acid, only the monomeric molecules and various adducts or cleavage products were detected at 0 V (monomeric ferulic acid (*m*/*z* 194.2 [M − H]^−^) or sinapinic acid (*m*/*z* 224.2 [M − H]^−^)). There were no dimeric or oligomeric products detectable above the limit of detection. Upon application of 1.8 V, an increase of the corresponding dimers (ferulic acid: *m*/*z* 389.1 [M − H]^−^; sinapinic acid: *m*/*z* 445.0 [M − H]^−^) and oligomeric products was recognized, analogously to chlorogenic acid and caffeic acid.

### 2.3. Investigation of Adduct Formation of Chlorogenic Acid and Alpha-Lactalbumin Using EC/LC/ESI-MS

**Figure 2:**
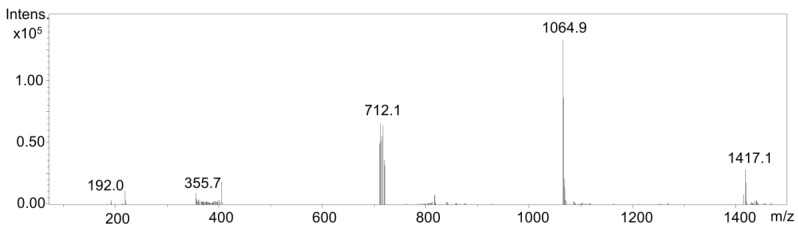
Full scan mass spectra at a retention time 13.5 min of oxidized chlorogenic acid.

For evaluating the reactivity of the electrochemically generated oxidation products of chlorogenic acid towards tryptic peptides of alpha-lactalbumin, the reaction of those two reactants was initiated via a reaction coil. Alpha-lactalbumin is a major component in mammalian milk. It is a whey protein with allergenic properties and, therefore, a very well-studied protein, and many epitopes have already been elucidated [17]. It is an acidic globular protein with a moderate size of 123 amino acids and a molecular mass of 14.2 kDa. The two-domain protein is stabilized by 4 disulfide bonds: The alpha-domain has disulfide bonds at positions Cys6-Cys120 and Cys28-Cys111, and the beta-domain has Cys61-Cys77 and Cys73-Cys91 disulfide bonds [18]. With its amino acids cysteine (in total 8) and lysine (in total 12), it provides several potentially reactive amino acid side chains, which could serve as binding sites for activated, electrophilic quinones or semiquinones. Defined volumes of both reactant solutions, phenolic acid and tryptic peptides, were injected to the electrochemical system via a syringe pump and a flow of 20 µL/min. Afterwards, the reaction products were directly transferred into an LC/ESI-MS system for chromatographic separation and detection. Negative ion mode in a scan range from 100 Da to 2000 Da was used to identify protein-phenol-adducts. Quinic acid, monomeric chlorogenic acid, and polymerization products were observed at a retention time of 13.5 min (*m*/*z* 192 [M − H]^−^ (quinic acid; 192 g/mol), *m*/*z* 354 [M − H]^−^ (monomeric chlorogenic acid; 354 g/mol), *m*/*z* 711 [M − H]^−^ (dimeric chlorogenic acid 711 g/mol), and *m*/*z* 1063 [M − H]^−^ (trimeric chlorogenic acid; 1063 g/mol) (Figure 2). It was even possible to detect the tetrameric derivative of chlorogenic acid with an *m*/*z* 1417 [M − H]^−^ (1417 g/mol)) (Figure 2).

**Figure 3:**
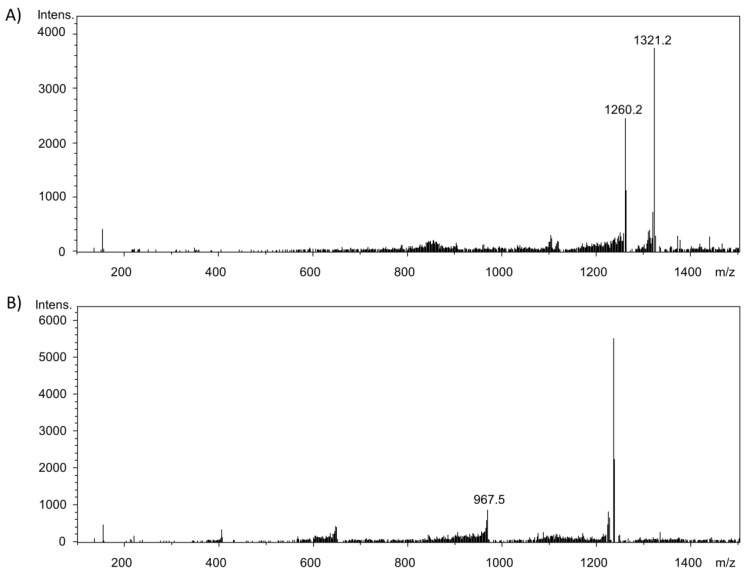
Full scan mass spectra after chromatographic separation via UPLC and detection using ESI-MS. (**A**) Potential adduct formation at retention time 14.6 min; (**B**) Potential adduct formation at retention time 18.5 min.

At 18.5 min, a signal with *m*/*z* 967.5 [M − H]^−^ was detected (Figure 3B), being assumed to be a reaction product of the monomeric form (*m*/*z* 354 [M − H]^−^) and one of the peptides (*m*/*z* 616 [M − H]^−^; Glu-Gln-Leu-Thr-Lys) of alpha-lactalbumin. This assignment can be supported by the fact that the peptide mentioned contains lysine residues and thus represents a very potent reaction partner. Furthermore, there were two signals at 14.6 min (*m*/*z* 1260.3 [M − H]^−^; *m*/*z* 1321.6 [M − H]^−^) (Figure 3A). The second signal correlates with an adduct formation between the dimer of chlorogenic acid (*m*/*z* 711 [M − H]^−^) and the peptide *m*/*z* 616 [M − H]^−^. In contrast, signal *m*/*z* 1260.3 [M − H]^−^ is different. It was hypothesized to be a condensation product of the peptides *m*/*z* 749 [M − H]^−^ and *m*/*z* 544 [M − H]^−^ due to their reactivity instead of a reaction product with a phenolic compound.

## 3. Discussion

Electrochemical systems provide a well-controlled application for generating activated metabolites and for further inducing redox-based adduct-formation. The prediction of the reaction mechanisms of highly reactive compounds is still challenging. Electrochemistry coupled to mass spectrometry is suitable to characterize optimum reaction conditions of the oxidation of phenolic compounds. Consequently, it is possible to generate starting material for follow-up reactions of the quinones that result. By controlling the reaction conditions and the use of on-line monitoring with, e.g., mass spectrometry, the adduct formation can be studied, and so far non-considered intermediates can be identified.

Phenolic compounds are known to be highly reactive. Several studies already shown an electrochemical oxidation of phenolic compounds such as caffeic acid and the formation of reactive intermediates [14,19]. In the present study, the structurally similar phenolic acids chlorogenic acid, caffeic acid, ferulic acid, and sinapinic acid were activated electrochemically for further adduct formation with proteins (or peptides thereof). The applicability of electrochemical oxidation by means of EC/ESI-MS concerning the four mentioned phenolic acids was successful. Oxidation products were investigated with regard to diversity and intensity in order to compare oxidative reactivities. Caffeic acid, as well as chlorogenic acid, are assigned to the group of hydroxycinnamic acids. These are characterized by an ortho-hydroxyl group and a conjugated double-bond in the side chain. They are able to form metastable radicals and reactive quinones and are therefore very accessible to nucleophilic attacks. As a result, an increased reactivity compared to further prominent phenolic acids is assumed. With regard to ferulic acid and sinapinic acid, a complete oxidation and formation of *o*-quinones is not possible with those chemical structures, as there is only one hydroxyl group. Instead of quinones, only semiquinones are formed [20]. As quinones are a key element of follow-up reactions of phenolic compounds, especially flavonoids [6], ferulic and sinapinic acid provide a lower reactivity than caffeic acid or chlorogenic acid. Proposed reactivities were confirmed by electrochemical application. The preference of phenolic compounds for oligomerization reactions was confirmed, as well. Various cleavage products and adducts were detected. The level of oligomerization depends on the structure of the phenolic compound. Caffeic acid and chlorogenic acid showed a higher degree of oligomerization and higher intensities than those of ferulic acid and sinapinic acid.

The reaction of the electrochemically oxidized products with peptides of a previously tryptically digested protein was performed in this study. This reaction was carried out exemplarily with chlorogenic acid and the peptides of the tryptically-digested protein alpha-lactalbumin. Previous research has already been successful in adjusting coupling reactions and generating adduct formation as well as protein labeling reactions [12,19,21]. Chlorogenic acid, an ester of caffeic and quinic acid, is a highly reactive secondary plant metabolite and is discussed as a potentially prevention of cardiovascular disease and because of its high antioxidant activity [22]. It is well known that chlorogenic acid tends to undergo oligomerization reactions prior to binding to other substrates such as biomolecules [23]. The use of the terms “monomer”, “dimer”, and “trimer” is not consistently synonymous with a regular and similar reaction of two or more of the exact initial substrate molecules in terms of oligomerization. With regard to the studies mentioned above, a reaction of two, three, or more initial phenolic molecules is assumed. Reactions can occur irregularly and differently and lead to several forms such as C-C or C-O bonds with a diverse bunch of leaving groups resulting in a variety of reaction products [24]. For instance, fragment of the monomeric chlorogenic acid is *m*/*z* 355.7 [M − H]^−^; the ‘dimer-like’ product is *m*/*z* 712.1 [M − H]^−^, providing a difference of *m*/*z* 356.4 [M − H]^−^. At this point, it is not clear to which extent the initial reactants undergo changes such as dehydration, oxidation, or binding to breakdown products prior to follow up reactions. Also, with regard to the further reaction partners (e.g., peptides), preliminary breakdown reactions or transformation cannot be ruled out. For example, chemical stability of peptides depends on the amino acid sequence and the side chains. Transformations such as hydrolysis, oxidation, or deamidation are possible. For this reason, irregularities in the mass differences of the reaction products may occur. Furthermore, data from mass spectrometric analysis may not lead to a structural elucidation, but rather give tentative indications.

According to the allergenic property of alpha-lactalbumin, it is of particular interest to study post-translational modifications of the protein, for example, resulting from a reaction with phenolic compounds. Therefore, it is necessary to investigate adduct formations with components of any kind and to investigate the effect on the binding properties of the epitope. Previous research has already investigated many reactions and their effect on the allergenicity of milk proteins such as phosphorylation [25].

**Figure 4:**
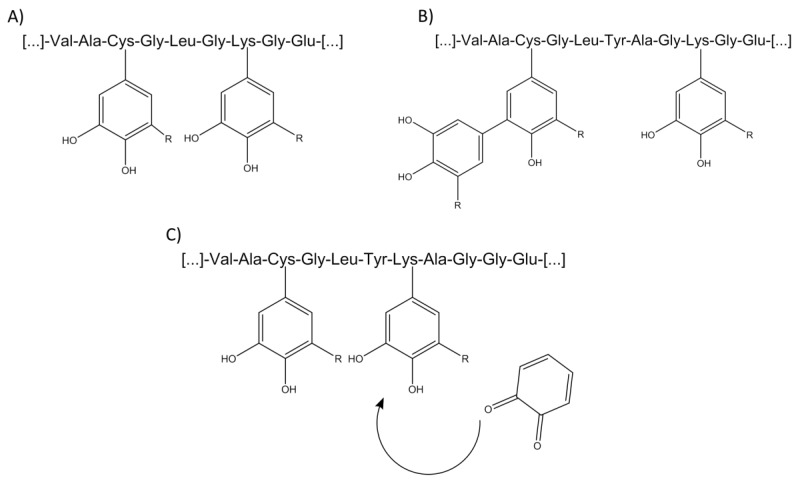
Varying reaction possibilities between peptides and phenolic compounds. (**A**) monomeric phenolic compound bound to a single amino acid residue of a peptide; (**B**) binding of different polymerization products of phenolic compounds to a peptide; (**C**) further reaction between bound phenolic compound with a further activated phenolic compound.

In order to evaluate adduct formation between phenolic compounds and further biomolecules, reaction mechanisms must be clarified. On the one hand, a simple reaction of a phenolic acid monomer respective of its quinoid structure with a reactive side chain of a peptide can take place (Figure 4A). Furthermore, it is also possible that not only monomers, but also polymerization products such as dimers, trimers, etc., can undergo reactions with the nucleophilic reaction partners (Figure 4B). However, it cannot be distinguished by mass spectrometry whether a reaction took place with either two monomers or a dimer when two reactive side chains are present in one protein/peptide. This issue can be seen in the case of the signal *m*/*z* 1321.6 [M − H]^−^ in the present study. Here, a reaction of a dimer of chlorogenic acid is assumed, but it could be obviously also a reaction of two monomers with the corresponding peptide. Even in this case, it is not clear if a reaction of the monomers can take place at different parts of the peptide at the same time or the monomer associated with the peptide reacts again with another phenolic compound in a subsequent step (Figure 4C). Previous research using size exclusion chromatography successfully demonstrated that phenolic acids seem to favor oligomerization reactions prior to binding to the proteins [26].

Besides the aspects mentioned so far, the elucidation of the interactions between phenolic compounds and proteins is still challenging due to the size and structure of the proteins. In previous studies, reactions between amino acids, peptides, or proteins, and reactive *o*-quinones or *o*-semiquinones, have been described [27,28,29]. In order to enable a better detection, enzymatic digestion of the target protein by using, e.g., trypsin, is recommended. Of note, the resulting peptides may also show crosslinking behavior. With regard to the detected signal *m*/*z* 1260.3 [M − H]^−^ in this study, it can be assumed that a reaction between two peptides occurred due to their reactivity.

The EC/LC/ESI-MS method introduced in this study indicated a significant contribution of the components to addition reactions. Many of the polymerization products could be assigned; three signals could be assigned to potential protein-phenol-adducts. However, many signals still could not be finally clarified at this time. Structural elucidation of the detected compounds by means of NMR is recommended. It is mandatory to isolate and separate the structures, for example, by means of preparative HPLC in advance. Due to the reactivities of the analytes, many signals were generated that could not yet be assigned. At this point, reactions between peptides next to peptide-phenol-interactions can be assumed. In comparison to the signals of the tryptic peptides of the protein digest, many previously detectable peptide signals of the tryptic digestion of alpha-lactalbumin could no longer be perceived with comparable intensity after the chromatographic separation of the electrochemically generated reaction products. This indicated that multiplicities of the tryptic peptides are significantly involved in reactions that may contribute to polymerization or adduct formation. Resulting from these investigations, it was shown that it is possible to produce and identify reaction products under controlled and defined conditions using electrochemistry coupled with mass spectrometry. Compared to traditional methods for the preparation of reaction products between phenolic compounds and peptides (pH value, temperature), reaction parameters could be optimized and the applicability of the electrochemical oxidation of phenolic compounds and subsequent coupling with another flow system to verify interactions could be demonstrated.

## 4. Materials and Methods

### 4.1. Chemicals

Sinapinic and ferulic acid were purchased from Carl Roth GmbH & Co. KG (Karlsruhe, Germany); caffeic acid, chlorogenic acid, alpha-lactalbumin (bovine), and ammonium acetate were purchased from Sigma Aldrich Chemie GmbH (Steinheim, Germany). Methanol and acetonitrile were purchased from Carl Roth GmbH & Co. KG (Karlsruhe, Germany). All chemicals were used in highest quality available. Water was purified before utilization via Direct-Q 3 UV-R system (Merck KGaA, Darmstadt, Germany).

### 4.2. Electrochemical Oxidation of Phenolic Acids by Means of EC/MS

**Figure 5:**
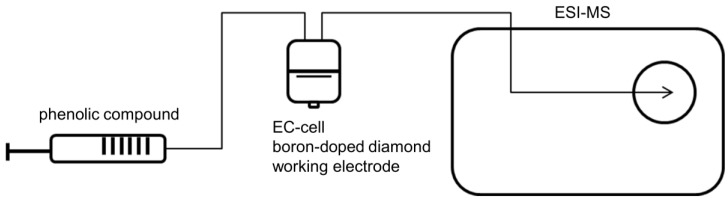
Instrumental setup for oxidation of phenolic acids by EC/ESI-MS. Phenolic acids are oxidized electrochemically via thin-layer cell including boron-doped diamond working electrode and are directly infused to ESI-MS.

Electrochemical oxidation of phenolic acids was performed using a preparative electrochemical thin-layer cell (µPrepCell, Antec, Leyden, Netherlands), consisting of a boron-doped diamond working electrode, a titanium counter electrode, and a Pd/H_2_ reference electrode. Potential was controlled using a Roxy^®^ potentiostat. A solution of 2 mM phenolic acid (in 90% MeOH (*v/v*) and 10% purified and double-distilled water (*v/v*) with 20 mM ammonium acetate) was injected into the electrochemical cell using a flow rate of 10 µL/min. Total volume of µ-prep cell is 11 µL depending on effective spacer thickness (here: 150 µm). Temperature of the electrochemical cell was set to 20 °C. A constant potential of 1.8 V was applied. Detection of oxidation products was performed with an ESI-MS ion trap mass analyzer in negative ion mode (amazon speed ETD, Bruker Daltonik GmbH, Bremen, Germany), with following mass spectrometer settings: ion spray voltage: 4.5 kV; ion source heater: 350 °C; source gas: 55 psi. For instrumental setup see Figure 5.

### 4.3 Tryptic Digestion of Alpha-Lactalbumin

Tryptic digestion of alpha-lactalbumin was performed with an aqueous solution of alpha-lactalbumin (Sigma Aldrich Chemie GmbH, Steinheim, Germany) in a concentration of 1 mg/mL protein (in purified and double-distilled water). It was digested tryptically (trypsin from porcine pancreas; Sigma Aldrich Chemie GmbH, Steinheim, Germany) for 16 h at 37 °C (protein-enzyme ratio 100:1). Digestion samples were used after SPE cleaning procedure via RP18ec SPR cartridges (Macherey Nagel GmbH & Co. KG, Düren, Germany) with 60% ACN for conditioning and washing steps and 0.2% aqueous formic acid for equilibration and elution steps. Afterwards, samples were dried using gaseous nitrogen and re-dissolved in a defined volume using 0.2% aqueous formic acid. Tryptic digest was analyzed using ESI-MS ion trap mass analyzer in negative ion mode (amazon speed ETD, Bruker Daltonik GmbH, Bremen, Germany), with following mass spectrometer settings: ion spray voltage: 4.5 kV; ion source heater: 350 °C; source gas: 55 psi. An assignment of signals and a resulting identification of the peptides were performed using the UniProtKB database (http://www.uniprot.org/) and the SIB Bioinformatics Resource Portal ExPASy (https://www.expasy.org/). Mentioned databases provide a comparison with the theoretical tryptic digestion of sequences. The peptides detected during this analysis are shown in Table 1.

### 4.4 Investigation of Adduct Formation of Chlorogenic Acid and Alpha-Lactalbumin Using EC/LC/ESI-MS

**Figure 6:**
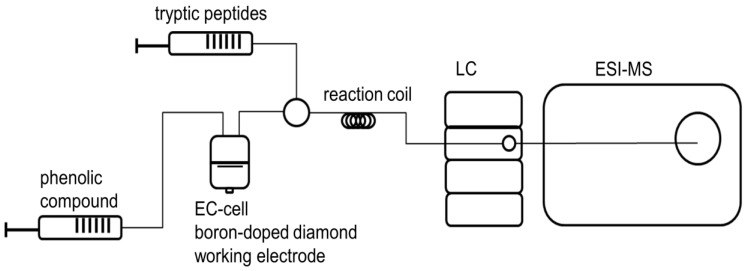
EC/LC/ESI-MS instrumental setup for oxidation, reaction, and separation of potentially phenol-protein-adducts. Phenolic acids are oxidized electrochemically via a thin-layer cell including a boron-doped diamond working electrode. A second flow system consists of a solution of tryptic peptides. Both solutions were combined using a three-way valve and a reaction coil and afterwards infused into the LC/ESI-MS system.

For considering adduct formation of oxidized chlorogenic acid and alpha-lactalbumin-deriven peptides, chlorogenic acid was oxidized as mentioned above (4.2). Subsequently, a second flow system was used to combine both analyte solutions. The ratio of both analytes in the reaction cell was 1:1 (*v/v*) regarding mentioned concentrations. A chromatographic separation was done on a Phenomenex^®^ (Phenomenex Inc., Torrance, CA, USA) reversed-phase HPLC column (Kinetex^®^ 2.6 µm RP 18 100 Å, 150 × 2.1mm) equipped with a guard column of the same material. A Dionex UltiMate™ 3000 UHPLC system (Thermo Fisher Scientific Inc., Waltham, MA, USA) was used. Detection was performed with a diode-array-detector as well as an ESI-MS ion trap mass analyzer (amazon speed ETD, Bruker Daltonik GmbH, Bremen, Germany), recording mass spectra in negative ion mode. The mobile phase A was water, and B was acetonitrile, both containing 0.1% formic acid. The gradient elution started with 95% A for 10 min, linearly increased to 60% B in 20 min and further up to 95% B in 3 min, and kept constant for 10 min. It was brought back to 95% A in 2 min followed by 10 min of re-equilibration. Injection volume was 1 µL using a flow rate of 200 µL/min for all samples. LC-MS system was controlled by HyStar 3.2 (Bruker Daltonik GmbH, Bremen, Germany). For instrumental setup see Figure 6.

## 5. Conclusions

Electrochemistry coupled with mass spectrometry was successfully used to investigate the redoxpotential of phenolic acids such as chlorogenic acid. It was possible to create optimized conditions for the oxidation of chlorogenic acid to generate a wide variety of oxidation products. Electrochemically oxidation of chlorogenic acid, caffeic acid, ferulic acid, and sinapinic acid generated a variety of reactive intermediates. Feasibility of this technique for the research topic was shown. Structural elucidation using NMR after isolation of generated oxidation products is planned in further studies to determine reaction mechanisms. The applicability of electrochemistry coupled with mass spectrometry for investigating reaction products of phenolic compounds and proteins could be demonstrated by the reaction of oxidized chlorogenic acid with peptides of alpha-lactalbumin. The method is suitable for generating adducts between proteins and phenolic components and is able to supplement or replace traditional methods. A detailed clarification of the resulting chemical structures of the products was not possible at this time. For this purpose, a synthesis of higher amounts of reaction products for an analysis with NMR is mandatory. With regard to a potential change of the protein allergenicity due to modification of the protein, methods such as ELISA, HPTLC-immunostaining, or HPTLC-aptastaining can be used [30,31].

## Figures and Tables

**Table 1:** Fragments, position in protein, and sequence of detectable peptides after tryptic digestion. Detection by use of high resolution mass spectrometry.

Fragments [*m*/*z*]	Position	Sequence
1198.6	118-127	Val-Gly-Ile-Asn-Tyr-Trp-Leu-Ala-His-Lys
749.4	113-118	Glu-Leu-Lys-Asp-Leu-Lys
652.3	25-29	Cys-Glu-Val-Phe-Arg
616.3	20-24	Glu-Gln-Leu-Thr-Lys
544.3	78-81	Ile-Trp-Cys-Lys
486.3	114-117	Ile-Leu-Asp-Lys
387.2	30-32	Glu-Leu-Lys
373.2	33-35	Asp-Leu-Lys
